# The Carboxy Terminus of the Ligand Peptide Determines the Stability of the MHC Class I Molecule H-2K^b^: A Combined Molecular Dynamics and Experimental Study

**DOI:** 10.1371/journal.pone.0135421

**Published:** 2015-08-13

**Authors:** Esam Tolba Abualrous, Sunil Kumar Saini, Venkat Raman Ramnarayan, Florin Tudor Ilca, Martin Zacharias, Sebastian Springer

**Affiliations:** 1 Department of Chemistry and Life Sciences, Jacobs University Bremen, Campus Ring 1, 28759 Bremen, Germany; 2 Department of Physics, Faculty of Science, Ain Shams University, Cairo, Egypt; 3 Physik-Department T38, Technische Universität München, James-Franck-Strasse 1, 85748 Garching, Germany; Johns Hopkins University, UNITED STATES

## Abstract

Major histocompatibility complex (MHC) class I molecules (proteins) bind peptides of eight to ten amino acids to present them at the cell surface to cytotoxic T cells. The class I binding groove binds the peptide via hydrogen bonds with the peptide termini and via diverse interactions with the anchor residue side chains of the peptide. To elucidate which of these interactions is most important for the thermodynamic and kinetic stability of the peptide-bound state, we have combined molecular dynamics simulations and experimental approaches in an investigation of the conformational dynamics and binding parameters of a murine class I molecule (H-2K^b^) with optimal and truncated natural peptide epitopes. We show that the F pocket region dominates the conformational and thermodynamic properties of the binding groove, and that therefore the binding of the C terminus of the peptide to the F pocket region plays a crucial role in bringing about the peptide-bound state of MHC class I.

## Introduction

**Fig 1:**
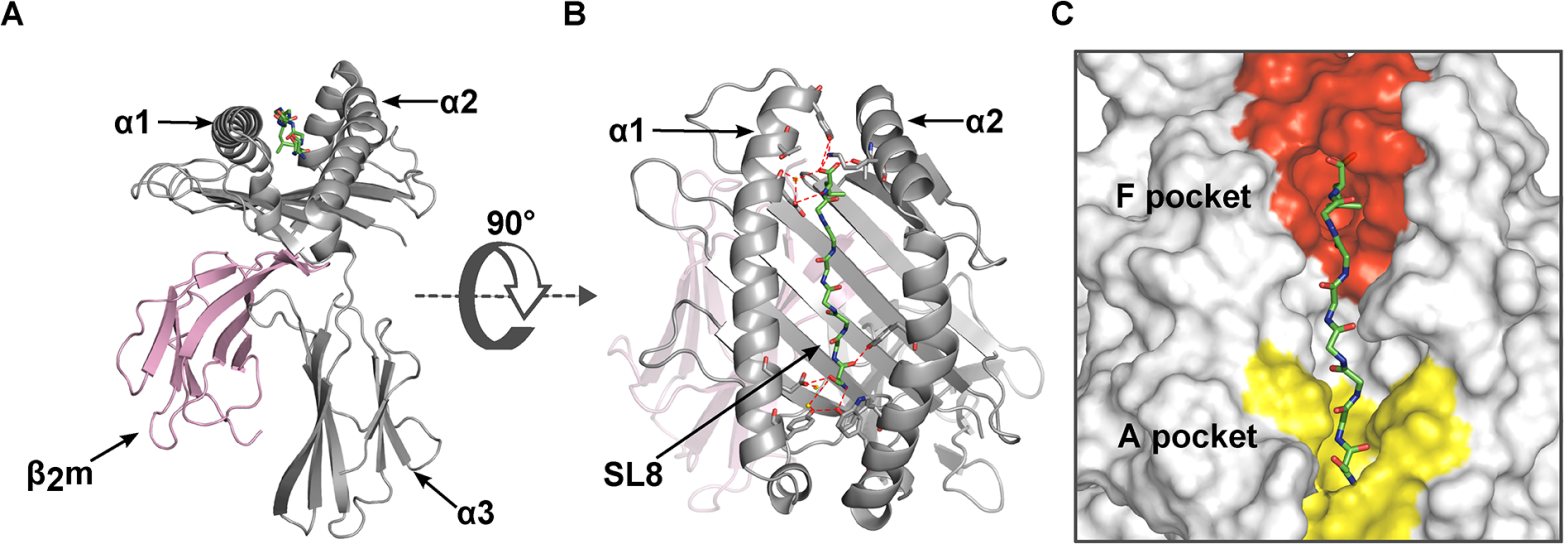
Crystal structure of the luminal domain of H-2K^b^. **(A)** Cartoon representation of class I heavy chain (gray) and the light chain β_2_m (pink). **(B)** Top view of the H-2K^b^ binding groove bound to an octamer antigenic peptide, SIINFEKL, represented as sticks of the peptide backbone (green carbon, red oxygen, and blue nitrogen). The hydrogen bonds between the peptide C and N termini and the binding groove are shown as red dashes. **(C)** The molecular surface of the F pocket (red) and the A pocket (yellow) of the H-2K^b^ binding groove (gray).

Major histocompatibility complex (MHC) class I molecules are transmembrane receptor proteins that transport intracellular peptides to the cell surface such that cytotoxic T cells can recognize epitopes of viral or tumor origin. The luminal part of an MHC class I heavy chain associates with the light chain beta-2 microglobulin (β_2_m) and then binds peptides in the endoplasmic reticulum (ER, Fig 1A). The stable ternary complex of heavy chain, β_2_m, and peptide then travels to the cell surface [1].

The peptide binding groove, formed by the α_1_/α_2_ superdomain, consists of an eight-stranded beta sheet platform topped by two alpha helices (Fig 1B) [2,3]. The groove is closed at both ends and usually accommodates a peptide of eight to ten amino acids only [4–6] that extends distinct side chains (called anchor residues) into defined pockets at the bottom of the groove. The peptide amino (N) and carboxy (C) termini form networks of hydrogen bonds in the regions around the A and F pockets at the ends of the groove (Fig 1C) in all class I/peptide complexes whose structure has been determined [7–9]. For stable binding to class I, optimal anchor residues are not strictly required [10–13].

In the cell, optimally loaded class I molecules are formed by selecting high-affinity peptides (*i*.*e*., peptides with a low dissociation rate) from the large pool of cellular peptides. This is central to the function of a class I molecule, since the complex must persist at the cell surface for hours to allow interactions with T cells [14]. The chaperone protein tapasin supports the selection of peptides in the ER that fulfill the specific length and sequence requirements for binding. There is no crystal structure of a tapasin-class I complex, but there is experimental and theoretical evidence that tapasin binds close to the F pocket [15–18].

To understand and eventually predict peptide selection in the cell, we will need to properly understand the binding energy contributions of each individual peptide-class I interaction, especially of the peptide termini. Empty class I molecules are suggested by several studies to be very flexible and energetically unstable [19–22]. Several lines of evidence suggest that in optimally loaded class I molecules, the restraint of the movements of the class I molecule by the peptide is a central feature of the peptide-bound state. To understand the energy contribution of the peptide interactions in stabilizing class I, Marlene Bouvier and Don Wiley first used circular dichroism to measure the midpoint of the thermal denaturation curve (T_m_, 'melting temperature') of HLA-A*02:01 folded *in vitro* in the presence of different truncated and modified peptides. Removal of the N-terminal amino group reduced the T_m_ by 21 K, whereas removal of the C-terminal carboxylate reduced it by 23 K. Based on this observation, they suggested that the interactions at C or N terminus peptide make the highest energetic contribution to the stability of the complex [20].

With the help of molecular dynamics (MD) simulations, we and others then showed that in murine and human class I molecules, the sections of the α_1_ and α_2_ helices around the F pocket that bind the peptide C terminus are conformationally flexible on a nanosecond time scale when no peptide is bound, whereas the A pocket region, which accommodates the N terminus of the peptide, is much more rigid and does not differ much in flexibility between the peptide-bound and peptide-empty states [23–26].

Experimental results support the role of the peptide C terminus in the conformational stabilization of class I [12]. A crystal structure of an empty class I molecule has not yet been obtained, but the B values of class I/peptide crystal structures show higher flexibility for the F pocket region than for the A pocket region [27–30]. The position of the F pocket region residues, especially the N terminus of the α_2_ helix, differs between crystal structures of the same class I allotype, suggesting a certain amount of adaptability of the F pocket region [23]. The crystal structure of H-2D^b^ with the pentapeptide NYPAL, which occupies the F but not the A pocket, is almost identical to that of D^b^ with full-length peptide [31]. With thermal denaturation experiments, we have shown that dipeptides that resemble the C termini of high-affinity peptides, and that presumably bind into the F pocket, help class I molecules to fold and protect them from denaturation [32].

In apparent contrast to these data, others have demonstrated that a 3_10_-helical fragment close to the A pocket changes conformation upon peptide binding to H-2L^d^, and they have proposed that this is the main conformational change in class I upon peptide binding [33]. Likewise, in our previous work, connecting the α_1_ and α_2_ helices by a disulfide bond close to the F pocket (which greatly restrains the mobility of this region in MD simulations) still allows normal chaperone interaction, peptide binding, and antigen presentation [34], suggesting that stabilization of the F pocket region by the peptide may not be essential for peptide binding to class I.

To precisely understand the contribution of individual functional groups of the peptide, especially the termini, to peptide binding, we have now combined MD simulations with biochemical and cellular approaches and studied the thermodynamic and conformational details of the binding of truncated and modified peptides. We find that the binding of the peptide C terminus to the F pocket is central to the conformational and thermal stability of the H-2K^b^/peptide complex. F pocket occupancy slows peptide dissociation and retains class I on the cell surface. Our work expands the molecular understanding of the details of the class I-peptide binding process, which will advance the ability to design vaccines for given peptides, and the development of small molecules that influence class I antigen presentation.

## Materials and Methods

### Molecular dynamics (MD) simulations

The crystal structures of H-2K^b^ in complex with the high-affinity peptides SIINFEKL and FAPGNYPAL (PDB ID code 1VAC [11] and 1KPV, respectively) served as the starting structure for the MD simulations, which were performed as in [35]. The detailed protocols are found in the supporting information.

### Thermal denaturation by tryptophan fluorescence (TDTF) measurements

TDTF measurements and T_m_ determination were performed exactly as described previously [36]. The detailed protocols are found in the supporting information.

### Half-maximal inhibitory concentration (IC_50_) measurements

Steady state measurements were performed with peptide-free folding reactions of K^b^/β_2_m. Following ultracentrifugation, different concentrations of full-length, truncated, or modified peptides were added for 5 min, and the competitive binding of 100 nM of the high affinity peptide SIINFEK_TAMRA_L, labeled with carboxytetramethylrhodamine (TAMRA) on the lysine side chain, was measured by anisotropy in the Cary fluorimeter with automated polarizers.

### Brefeldin A (BFA) decay assay

RMA-S cells [37] were kept overnight at 25°C and at t = 0 treated with 10 μg/ml BFA (Applichem or Alfa Aesar) and with 10 μM peptide and transferred to 37°C for a maximum of 4 hours. At different time points, cells were washed twice with 1x PBS/0.01% NaN_3_, and cell surface K^b^ was measured with the MAb Y3 by flow cytometry.

## Results and Discussion

### The peptide C terminus restrains the conformational dynamics of H-2K^b^

**Fig 2:**
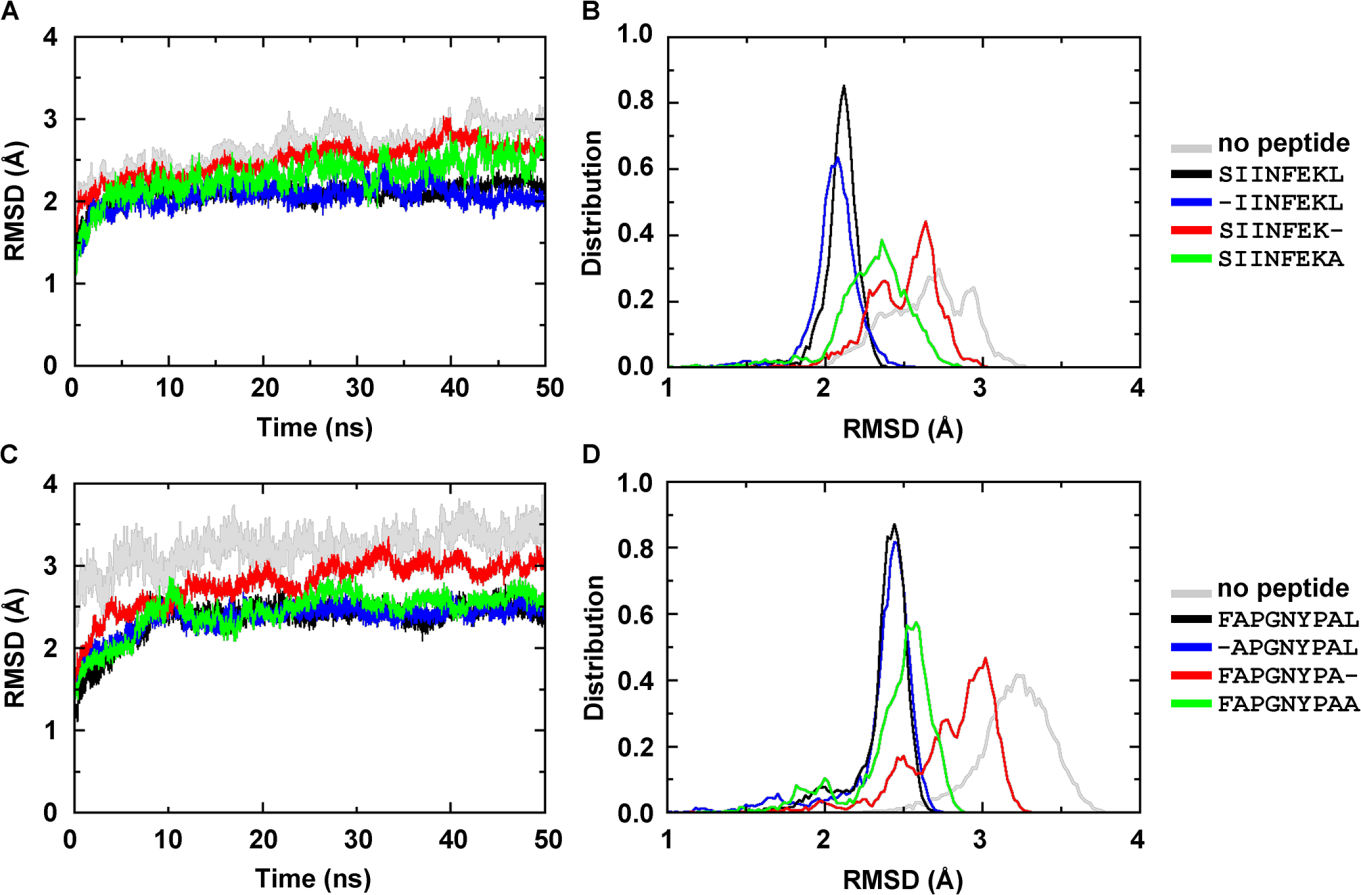
Root mean square deviation (RMSD) of the binding groove of H-2K^b^ (residues 1–180, gray), bound to the indicated peptides. The values were calculated from frames of two independent MD simulations. **(A)** RMSD time course for trajectories of the complexes derived from the K^b^/SIINFEKL crystal structure. **(B)** RMSD probability distribution of all trajectories for each molecule. **(C)** RMSD time course for the trajectories of the complexes derived from the K^b^/FAPGNYPAL crystal structure. **(D)** RMSD probability distribution calculated over the trajectories for each molecule.

We first investigated the effect of the terminal residues of the peptide on the conformational stability of the murine MHC class I allotype H-2K^b^ (K^b^) in MD simulations. As starting structures, we used published crystal structures of K^b^ bound to the high-affinity peptides SIINFEKL (K^b^/SIINFEKL; sequence in single-letter amino acid code) and FAPGNYPAL (K^b^/FAPGNYPAL) to construct models of K^b^ without peptide (empty: eK^b^_SIINFEKL_ and eK^b^_FAPGNYPAL_). We performed MD simulations with these four structures and measured the root mean square deviation (RMSD) for all residues that form the binding groove (res. 1–180). The binding grooves of both K^b^/SIINFEKL and K^b^/FAPGNYPAL showed narrow RMSD probability distribution peaks (Fig 2). Without peptide, in contrast, RMSD values were higher on average and more widely distributed, suggesting greater conformational fluctuation. Thus, as reported before [6,12,24,35,38,39], the total lack of peptide conformationally destabilized the binding groove.

To distinguish whether this destabilization was caused by the loss of binding of the peptide N terminus, the peptide C terminus, or both, we next performed and analyzed MD simulations of K^b^ bound to peptides that were truncated by a single residue at either end. When the C-terminal residue of the peptide (in the following abbreviated as Pω) was truncated (K^b^/SIINFEK and K^b^/FAPGNYPA), the RMSD values of the binding groove increased towards those of the empty binding groove. In surprising contrast, truncation of the peptide N terminus (K^b^/IINFEKL and K^b^/APGNYPAL) did not significantly affect the RMSD (Fig 2B and 2D). This suggests that binding of Pω is necessary to restrict the conformational motion of K^b^.

To differentiate between the stabilizing contributions of the carboxylate group and the Pω side chain, we simulated K^b^ complexes with peptides whose Pω was modified to alanine. The binding grooves of K^b^/SIINFEKA and K^b^/FAPGNYPAA complexes showed RMSD average values only slightly higher than those with the full-length peptide (with slightly broader peaks; Fig 2B and 2D; green). Impressively, however, when Pω of SIINFEKL was modified to glycine (SIINFEKG), entirely removing the side chain, we saw a significant increase in the RMSD, suggesting that even the small methyl group in the side chain of a C-terminal alanine helps stabilize the binding groove (S1A and S1B Fig).

When we removed the Pω carboxylate group of the peptide (replacing the leucine with isopentylamine to give the K^b^/SIINFEKL-Cdel complex), we saw similarly strong destabilization (S1A and S1B Fig). Thus, both the Pω side chain and the carboxylate are important to restrain the flexibility of the binding groove.

### The peptide C terminus restrains the mobility of the F pocket region

**Fig 3:**
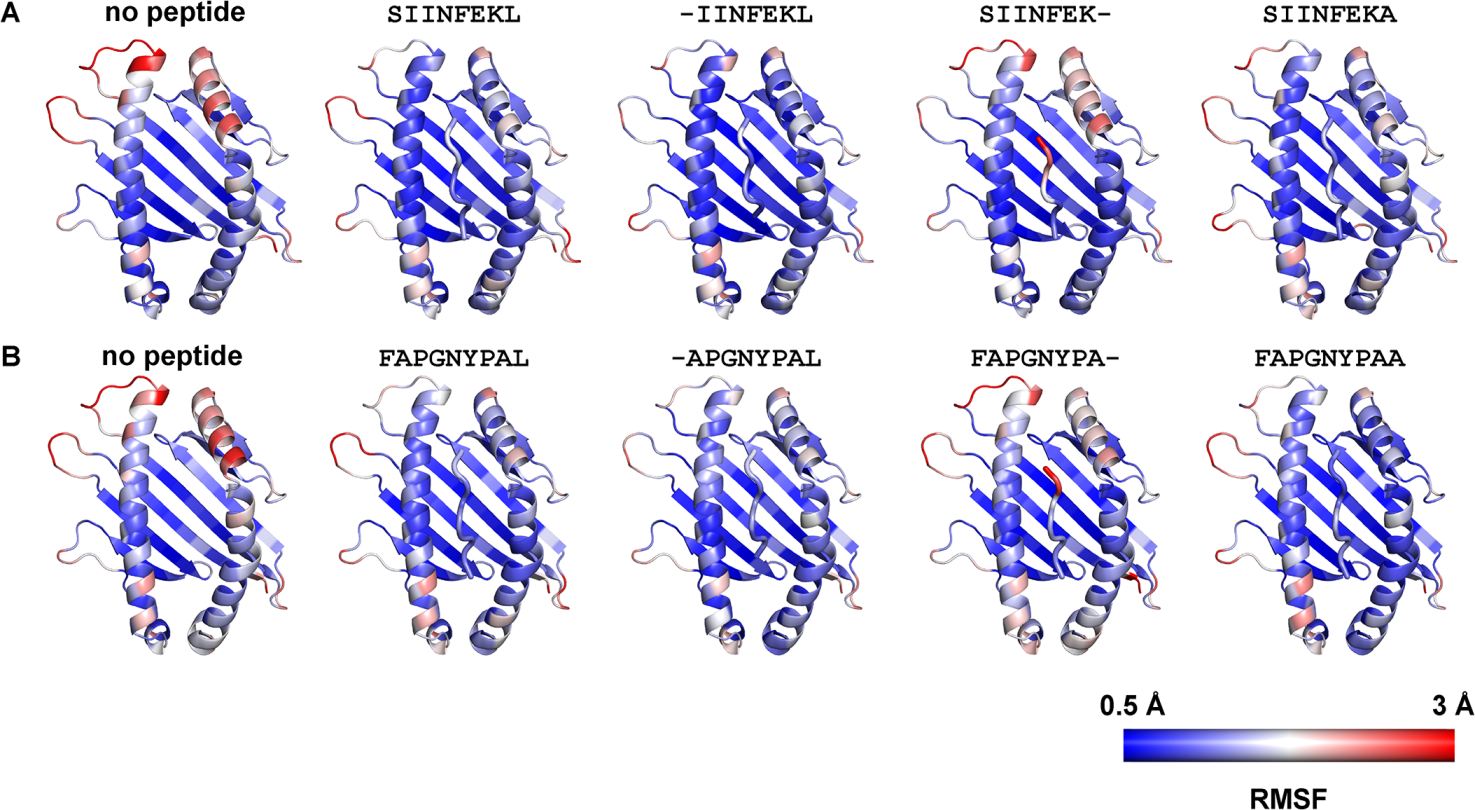
Color-coded view of the configurational flexibility of K^b^ binding groove and peptide calculated as root mean square fluctuations (RMSF) for each individual residue of the protein from two independent MD simulations of complexes with peptide derived from the (A) K^b^/SIINFEKL or (B) K^b^/FAPGNYPAL structures and depicted as a color (blue to red) on a cartoon representation of K^b^. The empty molecule and the K^b^/C-terminally truncated peptide complex show high flexibility of the alpha helices lining the F pocket.

To see which residues of the class I binding groove are conformationally stabilized by peptide binding, we next measured the root mean square fluctuation (RMSF) of each residue of the binding groove and colored the structure accordingly with a blue to red color spectrum (heat map; Fig 3 and S1C Fig). The binding grooves of the empty molecules were highly flexible, especially in the F pocket region, and the class I residues that bind the Pω showed the highest flexibility when the binding pocket was empty. Binding of the full-length peptides restrained the flexibility of these residues, but peptides that lacked Pω had no such restraining effect. For SIINFEKA, SIINFEKG and SIINFEKL-Cdel, the restraint was intermediate. In contrast, the K^b^ residues surrounding the A pocket always remained at similar levels of flexibility, whether without peptide, with full-length peptides, or with N- or C-terminally truncated peptides. Since the RMSF correlates with the configuration entropy ΔS_config_ (*i*.*e*., the entropy related to the position of the atoms rather than their velocity or momentum; [25, 26]), we conclude that in the peptide-empty state, the F pocket residues possess a high ΔS_config_ that is restrained upon peptide binding, whereas the remainder of the binding groove has a similar ΔS_config_ in both empty and peptide-bound structures.

### Binding free energy analysis shows a cooperative effect of the carboxylate group and the side chain

To more precisely quantify the role of the peptide N and C termini in the conformational stability of the K^b^/peptide complex, we calculated the free energy of peptide binding (ΔG) to the binding groove for both sets of MD simulations by combining the molecular mechanics force fields and the continuum solvation model [40–42] in the molecular mechanics Poisson-Boltzmann surface area (MM-PBSA) method (S1 Table). MM-PBSA performs well in measuring the relative values of receptor-ligand binding free energies [43–47]. It does not include the entropy term, which is very computationally demanding, but in earlier work, inclusion of the entropy term did not necessarily improve the prediction accuracy [46,48].

To rank the peptides based on their binding free energy, we then calculated the change in the binding free energy (ΔΔG_MM-PBSA_, S1 Table) that occurs upon modifying or truncating the peptide. The Pω truncations showed a much higher ΔΔG_MM-PBSA_ than the N-terminal truncations, suggesting a major contribution of the Pω to the peptide binding energy. Looking in more details, the change in ΔΔG_MM-PBSA_ was greater when the Pω carboxylate was removed than for the complexes with alanine- and glycine-modified C termini. This suggests an important contribution of the hydrogen bonds of the Pω carboxylate to the binding energy.

**Fig 4:**
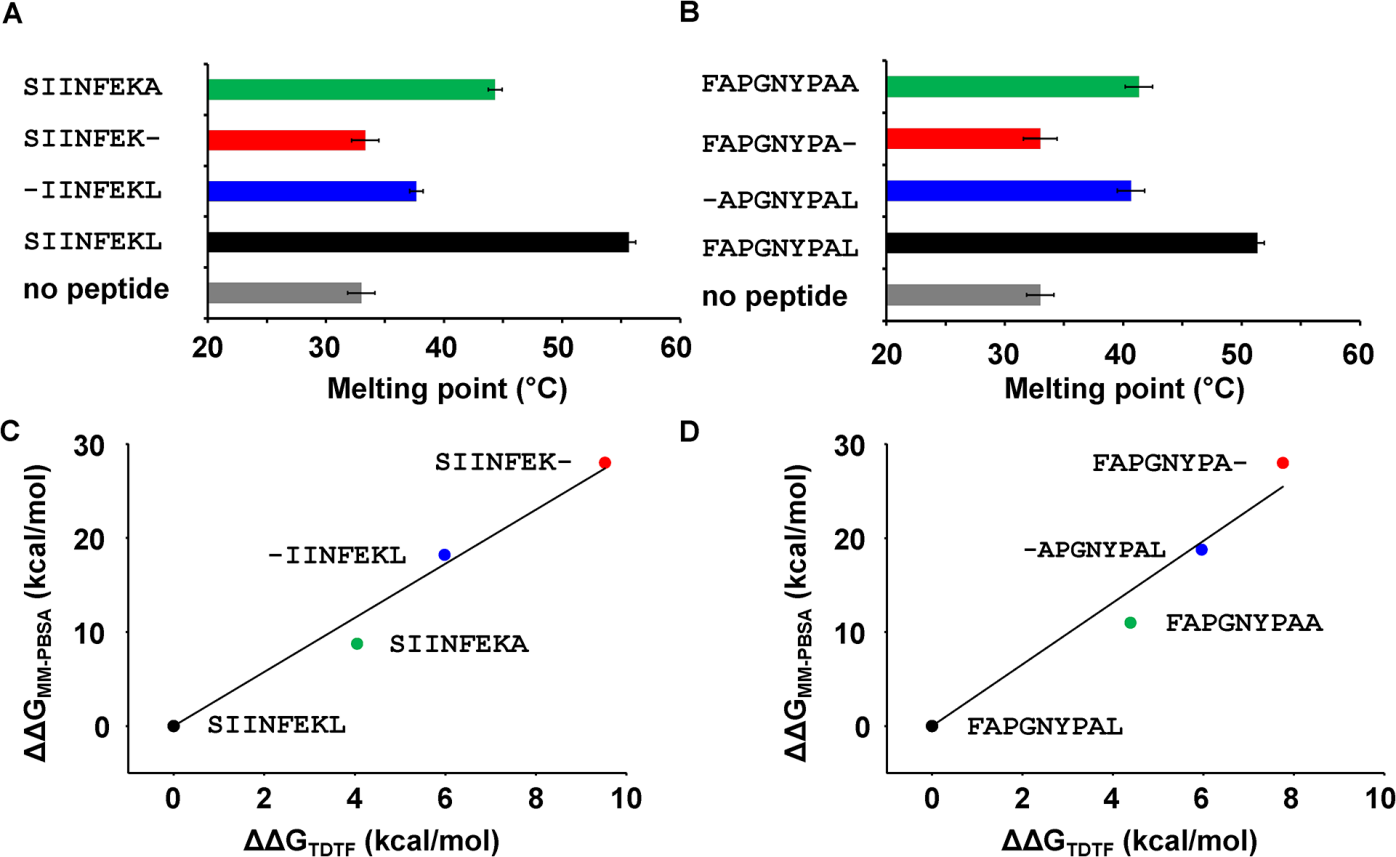
Thermal denaturation measured by tryptophan fluorescence (TDTF) shows the T_m_ of K^b^/β_2_m empty or in complex with peptide. **(A)** K^b^/β_2_m complexes folded empty or with SIINFEKL-derived peptide as indicated. **(B)** Analogous experiment, K^b^/β_2_m complexes with FAPGNYPAL-derived peptides. **(C) and (D)** correlations between ΔΔG values calculated from TDTF T_m_ (in A, B) and ΔΔG values from MM/PBSA (**S1 Table**).

To test our ranking by experiment, we next used the TDTF (thermal denaturation measured by tryptophan fluorescence) assay to determine the T_m_ of the K^b^/peptide complexes. In this assay, thermal unfolding of the class I molecule is measured by the decrease in fluorescence intensity that occurs when the tryptophan indoles are exposed to the aqueous environment [49,50]. The TDTF analysis was performed on the luminal domain of K^b^, which was produced in *E*.*coli* and folded from inclusion bodies as a complex with human β_2_m and with or without peptide [36]. The empty K^b^ molecule showed the lowest T_m_ (33°C), and binding of the full-length peptides increased the T_m_ dramatically (55.7°C for K^b^/SIINFEKL and 51.3°C for K^b^/FAPGNYPAL; Fig 4A and 4B and S1E Fig; and S2 Table). The complexes with alanine- and glycine-modified Pω, and those where the Pω carboxylate was removed, still showed some thermodynamic stabilization of K^b^. Such stabilization also occurred with N-terminal residue truncations, but with the Pω truncations K^b^/SIINFEK and K^b^/FAPGNYPA, no stabilization of the empty form at all was seen, as we reported before [36]. From the T_m_ values, we derived the ΔΔG_TDTF_ values using the two-state model of denaturation [50,51]. They correlate very well with the ΔΔ_MM-PBSA_ (correlation coefficients 0.97 and 0.95, Fig 4C and 4D).

Taken together, both theory and experimental data demonstrate in correlation a cooperative effect of the carboxylate group and the side chain at the C terminus of the peptide, which thus contributes much more to the binding energy and conformational stability of the K^b^/peptide complex than the N-terminal amino acid.

### Both C-terminal side chain and carboxylate are required for high binding affinity of the peptide

Next, we assessed the tendency of each modified peptide to become exchanged for a high-affinity peptide. We followed the hypothesis that greater flexibility of the binding groove in the MD simulations indicates a less perfect fit and thus correlates with lower binding affinity of the bound peptide. This was previously postulated for both class I and class II molecules [25,33,52,53].

**Fig 5:**
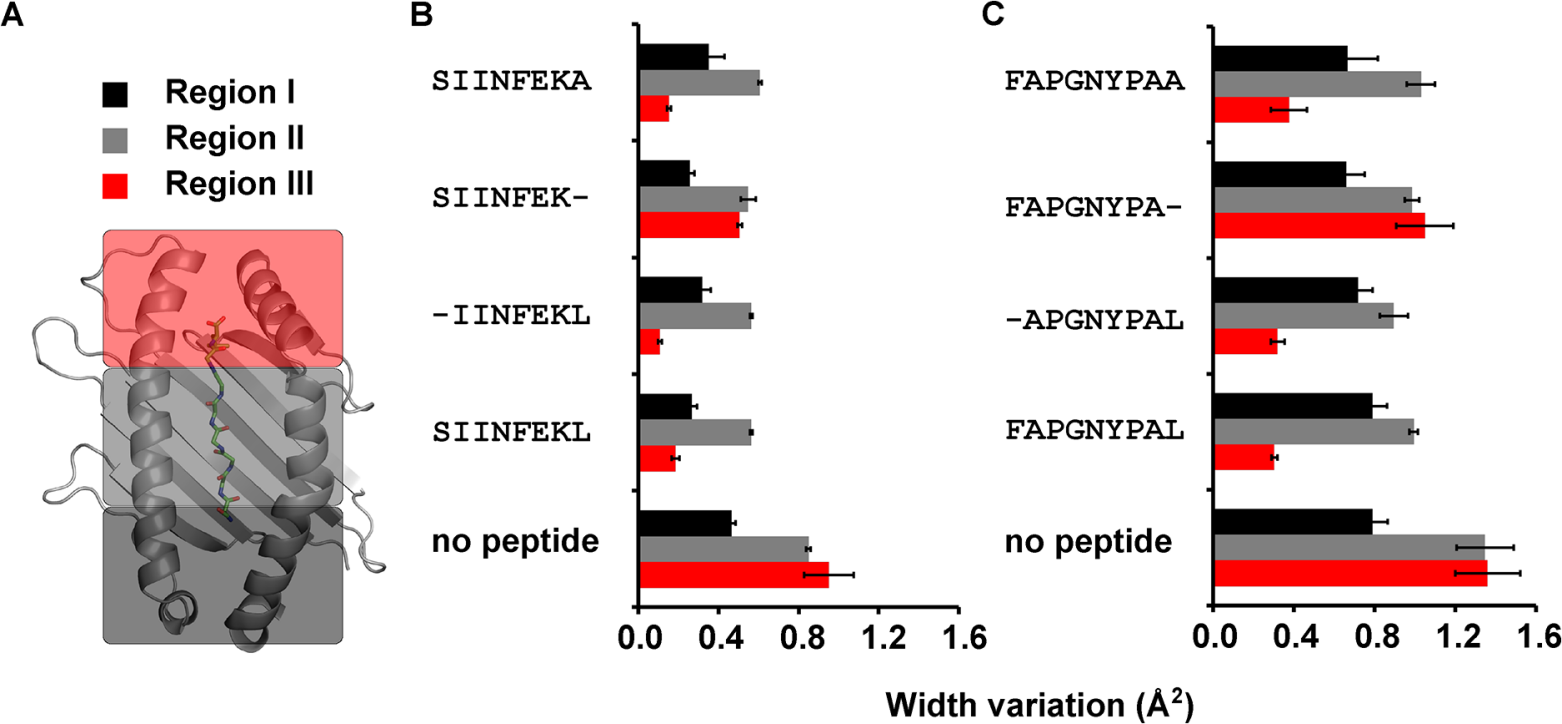
Variation of the binding groove width in MD simulations. **(A)** Region I (black, A pocket region, residues 50–59 and 165–176), Region II (gray, C pocket region, residues 60–72 and 152–164), and Region III (red, F pocket region, residues 73–84 and 139–150). **(B) and (C)** Width variance of each region measured as the distance of the centers of masses of the α carbons of respective opposing helical segments for complex derived from the SIINFEKL **(B)** and FAPGNYPAL **(C)** crystal structures. Error bars represent standard deviation.

To quantify the variation of the binding groove width on a local scale, we divided the binding groove into three regions (region I, which contains the A pocket, region II, in the center of the binding groove, and region III, which contains the F pocket; Fig 5A) and measured their width variation (WV) in the simulations. In all three regions, the WV was greatest when the binding groove was empty, similar to published data [54,55] (Fig 5B and 5C). With the full-length peptide bound, the WV decreased by ca. 0.2 Å^2^ in regions I and II compared to the empty molecule, but by a drastic 0.7 to 0.9 Å^2^ in region III. Thus, in agreement with the results shown in Fig 3, the bound peptide had the greatest stabilizing effect on the F pocket region.

The WV of region III for all complexes with truncated and mutated peptides was in the same range as for the full-length peptide complexes (Fig 5B and 5C, and S1E Fig), with the only exception of the Pω truncated peptides SIINFEK and FAPGNYP, which showed large WV, just like the empty K^b^ molecules. This shows that only the loss of the entire C-terminal amino acid causes a significant conformational fluctuation of the entire F pocket region.

**Fig 6:**
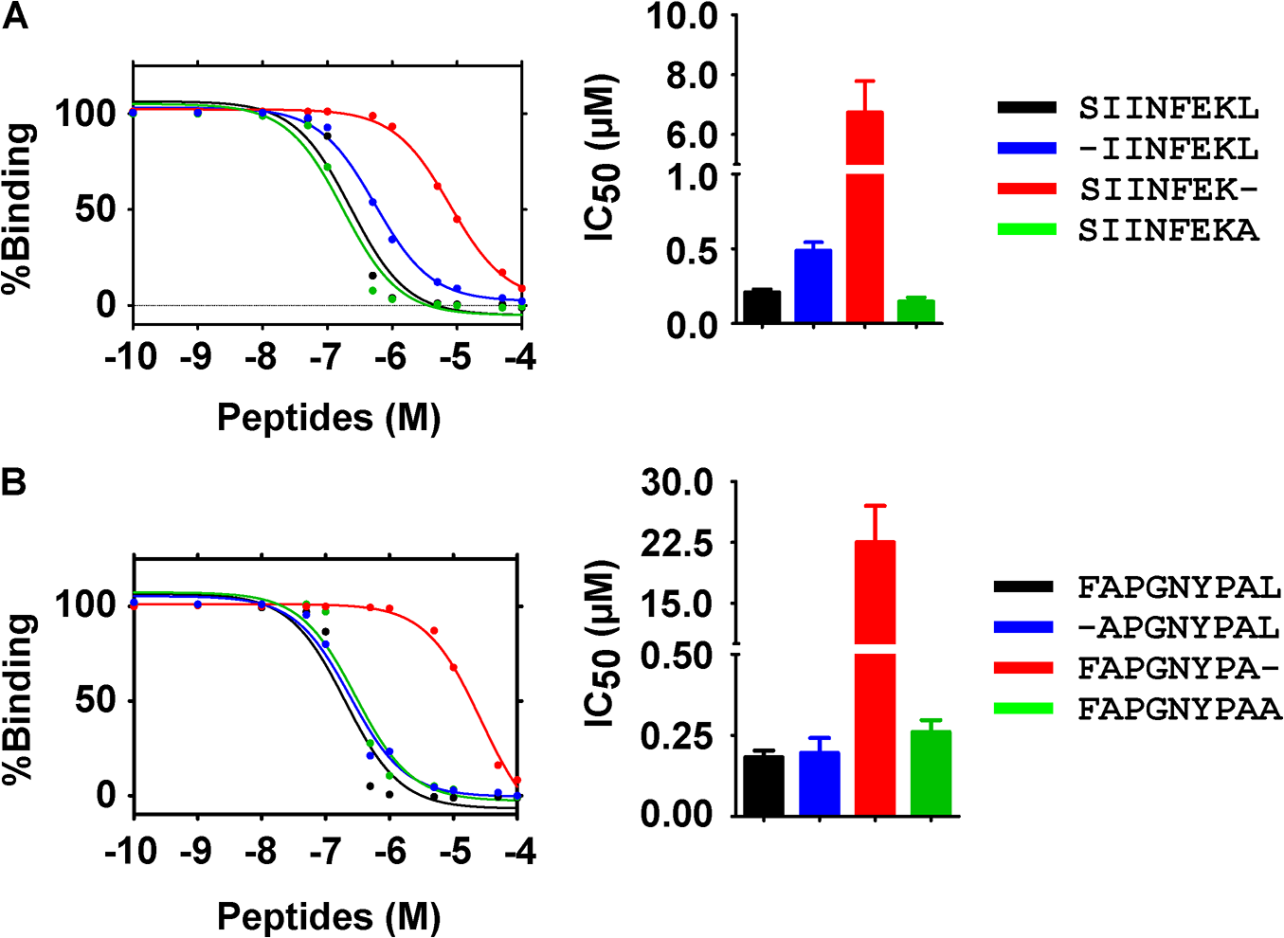
Half-maximal concentration of peptide (IC_50_) required to inhibit the binding of the high affinity peptide SIINFEK_TAMRA_L. K^b^/β_2_m complexes derived from the SIINFEKL (**A**) or the FAPGNYPAL (**B**) structures were folded empty and then incubated for 5 minutes with different concentrations of full-length, truncated, or modified peptides. The Pω truncated peptide shows the highest IC_50_ value. Numerical IC_50_ values: SIINFEKL, 0.19 μM; IINFEKL, 0.43 μM; SIINFEK, 7.7 μM; SIINFEKA, 0.12 μM; FAPGNYPAL, 0.16 μM; APGNYPAL, 0.15 μM; FAPGNYPA, 18.0 μM; FAPGNYPAA, 0.22 μM. Binding curves (left) are of one representative experiment, and the IC_50_ values (right) are the average of three experiments.

To test by laboratory experiment whether this conformational fluctuation indeed causes a lower complex stability of the Pω truncated peptides, we quantified the affinity of peptides to K^b^. K^b^ was folded empty [36] and then incubated with different concentrations of each peptide variant for five minutes. Then, the index peptide SIINFEK_TAMRA_L (fluorescently labeled on the lysine side chain) was added as a competitor, and the endpoint of its binding was measured by fluorescence anisotropy [56]. From theory curves fitted to the data, we calculated the half-maximal concentration of inhibition of SIINFEK_TAMRA_L binding (IC_50_) for each modified peptide (Fig 6, S1F Fig). We found that the IC_50_ values of nearly all peptide were similar, but those of the Pω truncated peptides SIINFEK and FAPGNYPA were one to two orders of magnitude higher. The experimental data confirm the conclusions from our MD simulations: the loss of the both the Pω carboxylate and the Pω side chain of the peptide destabilize the binding groove and thus facilitate dissociation of the bound peptide and subsequent binding of a new peptide.

### Engagement of the C terminus of the peptide retains class I molecules at the cell surface

**Fig 7:**
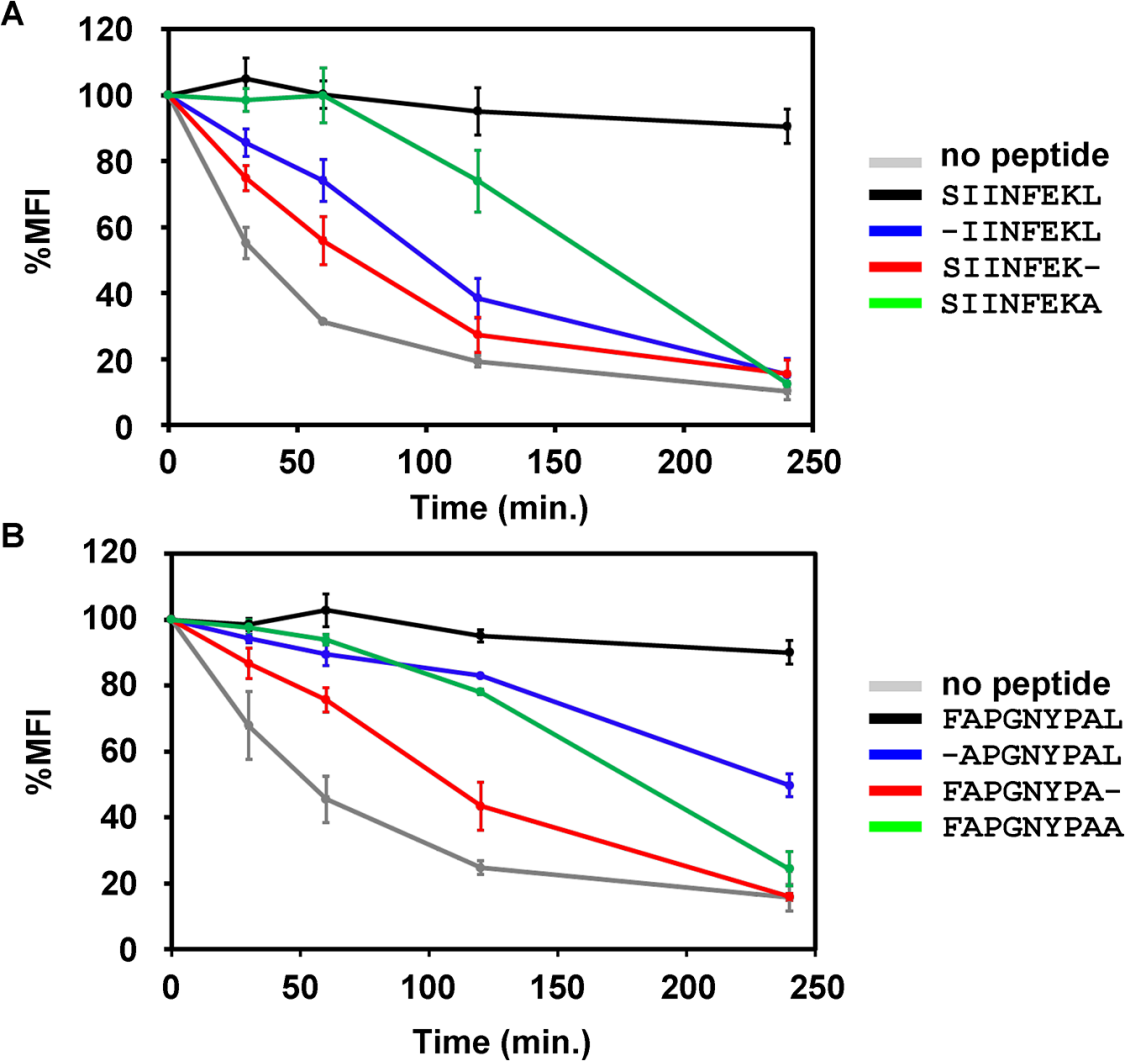
The K^b^/C-terminally truncated peptide complex shows the lowest stability on the cell surface in a BFA decay experiment. RMA-S cells were incubated overnight at 25°C, then 10 μM peptide (as indicated) was added to the medium, and cells were transferred to 37°C. H-2K^b^ surface levels were determined at each time point with MAb Y3 and flow cytometry. Averages ± SEM (n = 3) are normalized to initial mean fluorescence intensity (MFI).

Once at the cell surface, class I molecules are subject to a quality control–of unknown molecular mechanism–that results in rapid endocytic destruction of those complexes with low-affinity or no peptides [34]. To determine whether the role of the Pω in maintaining a stable class I/peptide complex also applies on the surface of live cells, we tested the stability of our K^b^/peptide complexes in a brefeldin A (BFA) decay experiment. Transporter associated with antigen processing (TAP)-deficient RMA-S cells, which cannot load high-affinity peptides onto K^b^, were kept overnight at 25°C to accumulate peptide-receptive class I molecules at the cell surface [37,57,58]. We then incubated these cells with each peptide and measured the surface residence time of the K^b^/peptide complexes by withdrawing aliquots of cells at different times, fixing, and staining with the antibody Y3 followed by flow cytometry (Fig 7, S1I Fig). As expected, more than 80% of the K^b^/SIINFEKL and K^b^/FAPGNYPAL complexes remained on the cell surface after four hours, whereas without peptide addition, about 50% of peptide-receptive K^b^ molecules were endocytosed after only 50 min of incubation. In comparison of the variant peptides, SIINFEK and FAPGNYPA showed the lowest efficiency in stabilizing K^b^ on the cell surface, whereas the Pω side chain and carboxylate deletions stabilized K^b^ to an intermediate extent. Thus, under the conditions of cell surface quality control, binding of the Pω is critically important for retaining class I at the surface.

### The free binding energy profile of the peptide C terminus in the F pocket depends on its side chain

**Fig 8:**
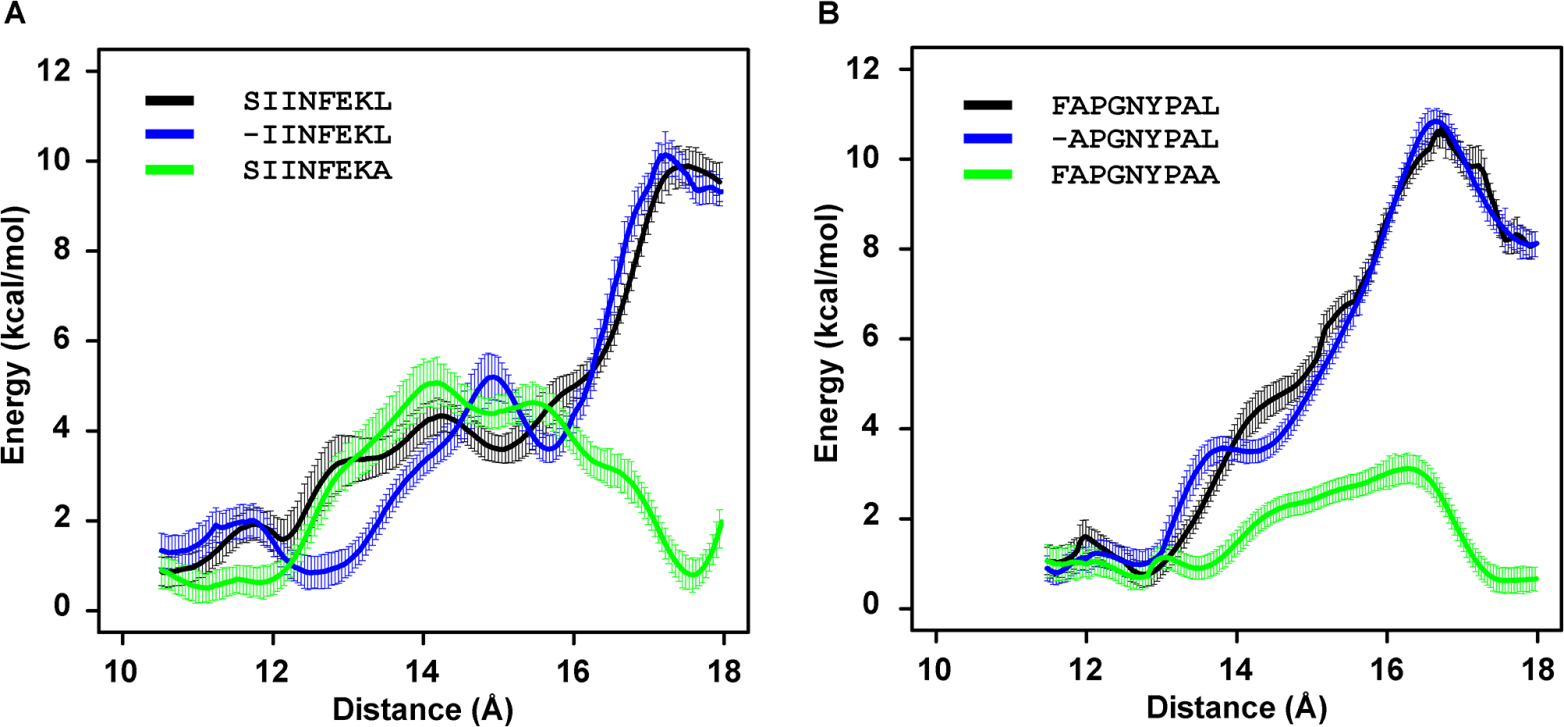
Calculated free energy change (potential of mean force, PMF) obtained from umbrella sampling simulations along the distance between the alpha carbon of the Pω and the bottom of the F pocket. Free energy changes were extracted from simulations of complexes with peptides derived from K^b^/SIINFEKL (**B**) or K^b^/FAPGNYPAL (**C**) structures. The start point and end point reaction coordinates are shown in the supporting material (S3 Fig).

Finally, we investigated in more detail the impact of altering the Pω side chain on the free energy profile of the peptide C terminus in the F pocket. We used umbrella sampling (US) simulations to compare the energy barriers along the dissociation pathway of the Pω (Fig 8A and 8B and S3 Fig). We simulated the K^b^/peptide complexes at different distance windows between the alpha carbon of the Pω and the bottom of the F pocket. Starting from the experimental crystal structure, the dissociation of the Pω from the F pocket was enforced, and weighted histogram analysis was employed to calculate the free energy profile along the reaction coordinate. The Pω leucine of the peptides SIINFEKL and IINFEKL had a free energy barrier that was ca. 11 kcal/mol higher than the Pω alanine or glycine (SIINFEKA, SIINFEKG) at a distance of 17 Å from the bottom of the F pocket, whereas the truncation of the Pω carboxylate (SIINFEKL-Cdel) showed ca. 5 kcal/mol at the same distance (Fig 8A, S1G Fig). In a similar manner, the Pω leucine in both FAPGNYPAL and APGNYPAL had a free energy barrier of ca. 8 kcal/mol higher than the Pω alanine (FAPGNYPAA) at a distance 16 Å from the bottom of the F pocket (Fig 8B). These data suggest that the F pocket of K^b^ requires both a long bulky side chain [59,60] and the Pω carboxylate for tight binding of the C terminus of the peptide.

## Discussion and Conclusions

Many attempts have been made to describe the molecular details of peptide binding to class I [7,9,12,55,61]. The interactions that occur between the peptide termini and the residues in the A and F pocket make the major energy contribution to binding [9,12,61,62]. Binding of the peptide N terminus into the A pocket is mainly via a network of hydrogen bonds with a group of conserved tyrosine residues [11]. At the other end of the binding groove, the C terminus of the peptide binds to residues in the F pocket region, also via hydrogen bonds [12]. The orientation of the peptide allows the Pω side chain to be buried deeply within the F pocket (Fig 1B).

Most previous studies of peptide binding to class I only compare optimally loaded class I molecules with empty ones [24,25,53,63]. However, suboptimally loaded class I molecules have thermodynamic and conformational properties that yield a better understanding of peptide binding [19,31,32]. In this study, we have performed MD simulations of K^b^ molecules bound to natural full-length peptides and to their suboptimal variants that were truncated or altered at their termini in order to determine the effect of peptide truncations on the dynamics and binding energy of the class I peptide binding groove. Since no crystal structures of empty or suboptimally loaded class I molecules are available (except for D^b^/NYPAL, [31], we started all simulations with the crystal structures of the high-affinity peptide complexes K^b^/SIINFEKL and K^b^/FAPGNYPAL that we modified to obtain the peptide truncations. To minimize the risk that the trajectories might be guided by the starting structure, we used two different starting crystal structures (see the materials and methods), and we energy-minimized and equilibrated all structures before starting the simulations. Our simulations cannot fully ensure that in the test tube, the short peptides bind in the same register to class I as the full-length peptides, but the closed ends of the class I binding groove and its topology make it likely.

Our MD simulations show a greater conformational stability for optimally loaded complexes compared to empty class I molecules, which confirms the need for the full-length peptide to restrain the conformational motions of K^b^ binding groove [23–25]. Interestingly, and in contrast to published data for the murine allotype H-2L^d^ [33], the complexes with truncated peptide N terminus show the same degree of conformational restriction as with full length peptide, whereas truncation of the peptide C terminus results in high flexibility of the F pocket region, similar to that of the empty groove (Figs 2 and 3). Our results are consistent with previous MD simulation studies of human and murine allotypes (H-2D^b^, H-2K^b^ and HLA-B*27:05) [23,24,32,39] and suggest that the binding of the Pω to the flexible F pocket region is required to restrain such flexibility and stabilize the class I binding groove. This major role of the F pocket region in the stability of the K^b^/peptide complex is confirmed by the TDTF results (Fig 4A and 4B). We have shown previously that upon heating of a complex of K^b^ with a suboptimal peptide, the peptide dissociates quickly from the binding groove, and the empty class I denatures [36].

If the complex of K^b^ with C-terminally truncated peptides is less resistant to heat, it might also show a higher dissociation rate at room temperature. Indeed, the IC_50_ values in Fig 6 strongly point to a major role of the Pω in maintaining peptide binding to class I, indicating that as long as the F pocket is occupied by the Pω, it is difficult to exchange the bound peptide. This is especially important since peptide dissociation co-determines the peptide exchange and optimization in the cell [64–66]. On the cell surface, truncation of the Pω increases the endocytosis rate of class I (Fig 7). This is probably because of the drastic decrease in the ΔG of the complex (S1 Table).

Our data suggest a common model where the peptide binding groove of K^b^ has two dynamic states, peptide-bound and peptide-empty. The biggest difference between these states is visible in the F pocket region. In the peptide-bound state, the helices of the F pocket region are rigid, and their distance is constant. In the peptide-empty state, the helices of the F pocket region are very flexible, and their distance fluctuates. It has been shown experimentally that the helices may partially unfold on a longer time scale [67]. We have found that binding of the Pω triggers the transition between the peptide-empty and the peptide-bound states and thus determines the peptide-bound conformation of the entire class I molecule.

Our data reveal a synergistic effect of the carboxylate group and the Pω side chain. Compared to the effect of the removal of the Pω, the truncations of either the carboxylate group or the side chain show an intermediate effect on the conformational and thermal stability of the complex. Thus, we suggest that this role of the Pω in the F pocket region follows an entropy-enthalpy compensation pathway, as observed previously for the binding between class I and the T cell receptor [68–72]. The binding enthalpy increases via a network of hydrogen bonds with the peptide C terminus, whereas the entropy is restrained by the binding of the Pω side chain to the bottom of the F pocket.

The prominent role of the F pocket region in our model is consistent with binding of tapasin close to the F pocket region of class I to mediate thermodynamic stabilization of class I as well as peptide exchange and optimization, as suggested previously [15–17,19,21,73,74]. We assume that tapasin helps to structure the F pocket region of a suboptimally loaded class I molecule such that it can bind optimal peptide [39].

Our data support the notion that short peptides and similar small compounds can be used to stabilize class I proteins on the cell surface in a peptide-receptive conformation. This is especially important for efficient T cell activation and improved vaccination. It also opens a window for the modelling of small compounds to bind specifically to the F pocket region and thus enhance or inhibit the binding of the peptide ensemble in a predefined conformation.

In agreement with previous MD studies, which have shown that the dynamics of different domains in class I and other proteins might be coupled [25,75], the findings in this work enable further analysis of the impact of the F pocket region on the global dynamics of other class I domains and the entire protein. Our study also illustrates that MD simulations can be complemented by experimental data to describe the mechanism of ligand-receptor binding and to provide atomic-level details that help in understanding its molecular basis.

## Supporting Information

S1 Fig (A) RMSD time course for trajectories of the complexes derived from the K^b^/SIINFEKL crystal structure. (B) RMSD probability distribution of all trajectories for each molecule eK^b^SIINFEKL shows four distinct peaks (approx. 2.3, 2.6, 2.8, and 3.2 Å), whereas K^b^/SIINFEKL show narrow peaks at 2.2 Å. K^b^/SIINFEKG shows broad peak at 2.8 Å and K^b^/SIINFEKL-Cdel shows two peaks at 2.5 and 3 Å. (C) Color-coded view of the configurational flexibility of K^b^ binding groove and peptide calculated as root mean square fluctuations (RMSF) for each individual residue of the protein from MD simulations of peptide complexes K^b^/SIINFEKG and K^b^/SIINFEKL-Cdel. (D) Variation of the binding groove width in MD simulations. A: Region I (black, A pocket region, residues 50–59 and 165–176), Region II (gray, C pocket region, residues 60–72 and 152–164), and Region III (red, F pocket region, residues 73–84 and 139–150). (E) Thermal denaturation measured by tryptophan fluorescence (TDTF) shows the T_m_ of K^b^/β_2_m empty or in complex with peptide. (F) Half-maximal concentration of peptide (IC_50_) required to inhibit the binding of the high affinity peptide SIINFEK_TAMRA_L. (G) Calculated free energy change (potential of mean force, PMF) obtained from umbrella sampling simulations along the distance between the Pω alpha carbon and the bottom of the F pocket. Free energy changes were extracted from simulations of complexes with peptides as indicated. (H) BFA decay experiment performed with RMA-S cells. H-2K^b^ surface levels were determined at each time point with MAb Y3 and flow cytometry. Averages ± SEM (n = 3) are normalized to initial mean fluorescence intensity (MFI).
(DOCX)

S2 Fig Number of clusters as a function of cumulative simulation time. **(A)** complexes with SIINFEKL-derived peptides **(B)** complexes with FAPGNYPAL-derived peptides.
(DOCX)

S3 Fig Representative snapshots of the start and end of the reaction coordinates used for the calculations of the free energy change (potential of mean force, PMF) obtained from umbrella sampling simulations along the distance between the Pω alpha carbone and the bottom of the F pocket. (DOCX)

S4 Fig Thermal denaturation of low-affinity peptide complexes is dependent of the concentration of free peptide. TDTF experiments were performed with the H-2K^b^-hβ2m-peptide complex in the presence of different peptide concentration. The Tm values of low-affinity peptide complexes increase with the free peptide concentration, but the relative difference in the T_m_ values between the C-terminal and N-terminal truncation remains.
(DOCX)

S1 Protocol Detailed Protocols. (DOCX)

S1 Table Experimental and calculated binding free energy (kcal/mol) using MM-PBSA and TDTF methods for H-2K^b^/peptide complexes. The error is calculated as standard deviations over the defined clusters.
(DOCX)

S2 Table Thermal denaturation measured by tryptophan fluorescence (TDTF) shows the T_m_ of K^b^/β_2_m empty or in complex with peptide, as indicated. The error is calculated as standard deviation.
(DOCX)
