# Supplementary material for: The Carboxy Terminus of the Ligand Peptide Determines the Stability of the MHC Class I Molecule H-2Kb: A Combined Molecular Dynamics and Experimental Study
Source: PLoS One. 2015 Aug 13;10(8):e0135421. doi: 10.1371/journal.pone.0135421 (PMC4535769; doi:10.1371/journal.pone.0135421)
Supplement: S1 Fig — H-2Kb surface levels were determined at each time point with MAb Y3 and flow cytometry. Averages ± SEM (n = 3) are normalized to initial mean fluorescence intensity (MFI). (DOCX) [file pone.0135421.s001.docx]

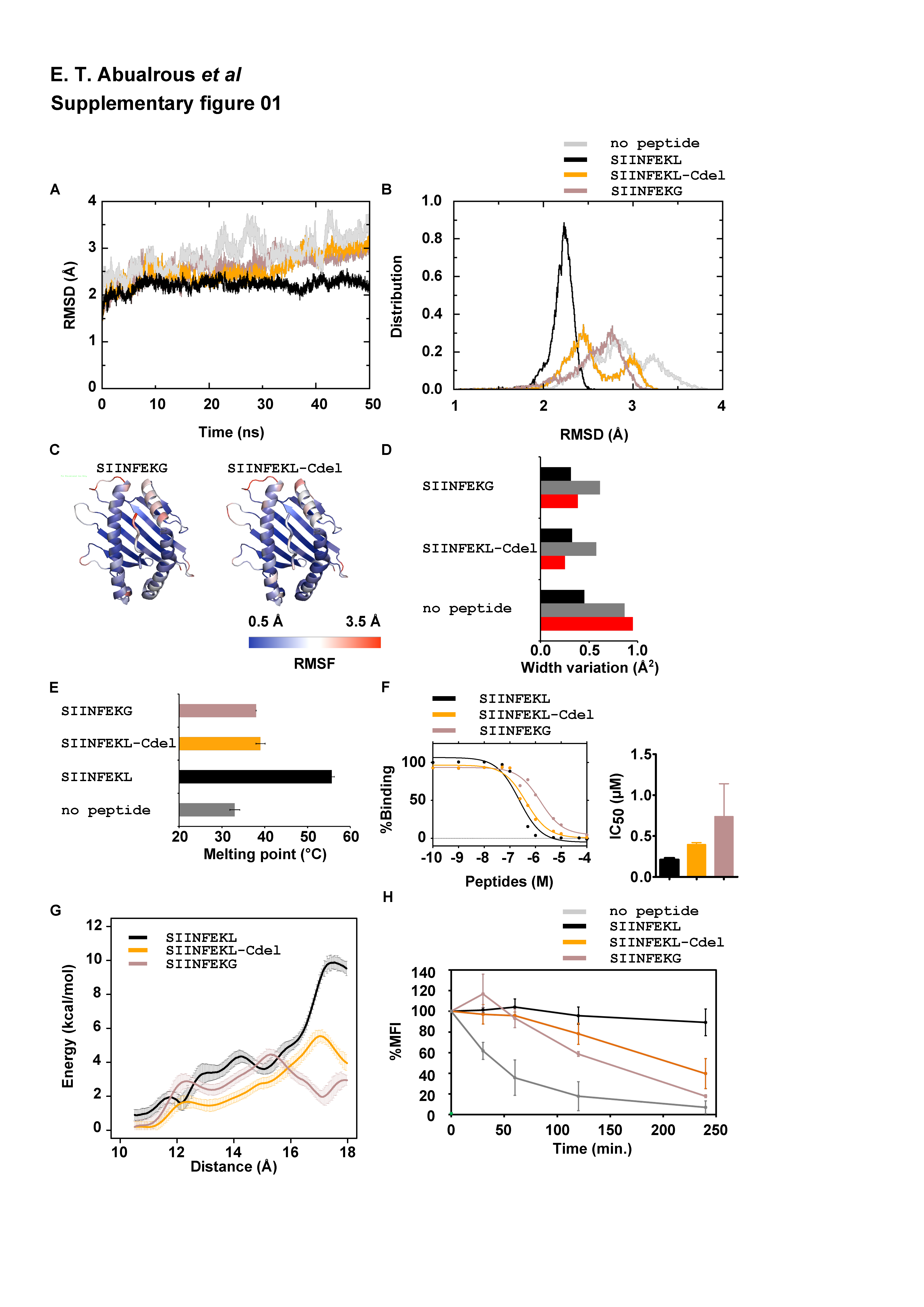


**S1 Fig. (A)** RMSD time course for trajectories of the complexes derived from the K^b^/SIINFEKL crystal structure. **(B)** RMSD probability distribution of all trajectories for each molecule eK^b^_SIINFEKL_ shows four distinct peaks (approx. 2.3, 2.6, 2.8, and 3.2 Å), whereas K^b^/SIINFEKL show narrow peaks at 2.2 Å. K^b^/SIINFEKG shows broad peak at 2.8 Å and K^b^/SIINFEKL‑Cdel shows two peaks at 2.5 and 3 Å. **(C)** Color‑coded view of the configurational flexibility of K^b^ binding groove and peptide calculated as root mean square fluctuations (RMSF) for each individual residue of the protein from MD simulations of peptide complexes K^b^/SIINFEKG and K^b^/SIINFEKL‑Cdel. **(D)** Variation of the binding groove width in MD simulations. A: Region I (black, A pocket region, residues 50-59 and 165-176), Region II (gray, C pocket region, residues 60-72 and 152-164), and Region III (red, F pocket region, residues 73-84 and 139-150). **(E)** Thermal denaturation measured by tryptophan fluorescence (TDTF) shows the T_m_ of K^b^/β_2_m empty or in complex with peptide. **(F)** Half-maximal concentration of peptide (IC_50_) required to inhibit the binding of the high affinity peptide SIINFEK_TAMRA_L. **(G)** Calculated free energy change (potential of mean force, PMF) obtained from umbrella sampling simulations along the distance between the alpha carbon of Pω and the bottom of the F pocket. Free energy changes were extracted from simulations of complexes with peptides as indicated. **(H)** BFA decay experiment performed with RMA‑S cells. H‑2K^b^ surface levels were determined at each time point with MAb Y3 and flow cytometry. Averages ± SEM (n = 3) are normalized to initial mean fluorescence intensity (MFI).
