# Supplementary material for: The Carboxy Terminus of the Ligand Peptide Determines the Stability of the MHC Class I Molecule H-2Kb: A Combined Molecular Dynamics and Experimental Study
Source: PLoS One. 2015 Aug 13;10(8):e0135421. doi: 10.1371/journal.pone.0135421 (PMC4535769; doi:10.1371/journal.pone.0135421)
Supplement: S2 Fig — (A) complexes with SIINFEKL-derived peptides (B) complexes with FAPGNYPAL-derived peptides. (DOCX) [file pone.0135421.s002.docx]

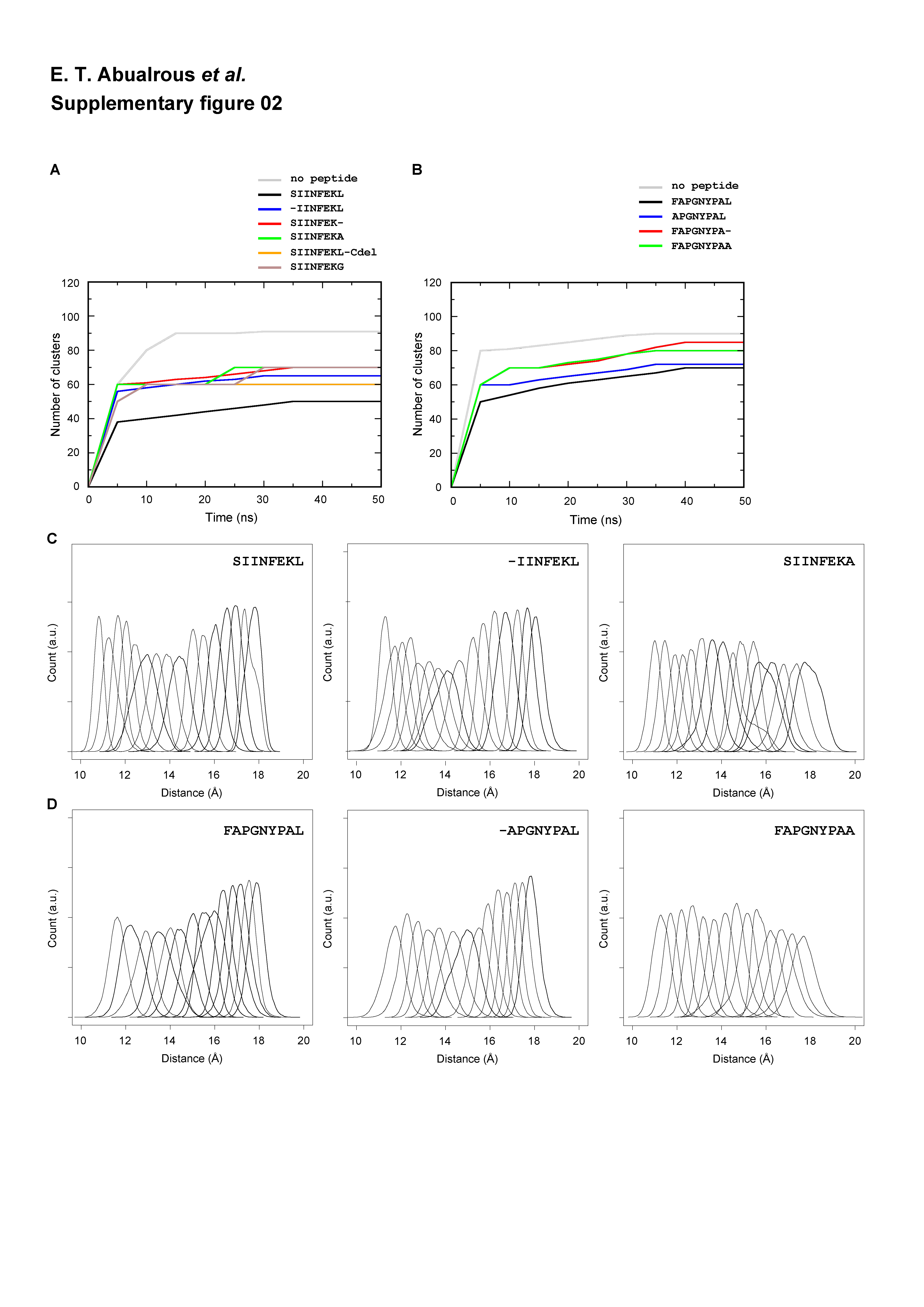


**S2 Fig. Number of clusters** as a function of cumulative simulations time of **(A)** complexes with SIINFEKL-derived peptides **(B)** complexes with FAPGNYPAL-derived peptides.
