# Supplementary material for: The Carboxy Terminus of the Ligand Peptide Determines the Stability of the MHC Class I Molecule H-2Kb: A Combined Molecular Dynamics and Experimental Study
Source: PLoS One. 2015 Aug 13;10(8):e0135421. doi: 10.1371/journal.pone.0135421 (PMC4535769; doi:10.1371/journal.pone.0135421)
Supplement: S3 Fig — (DOCX) [file pone.0135421.s003.docx]

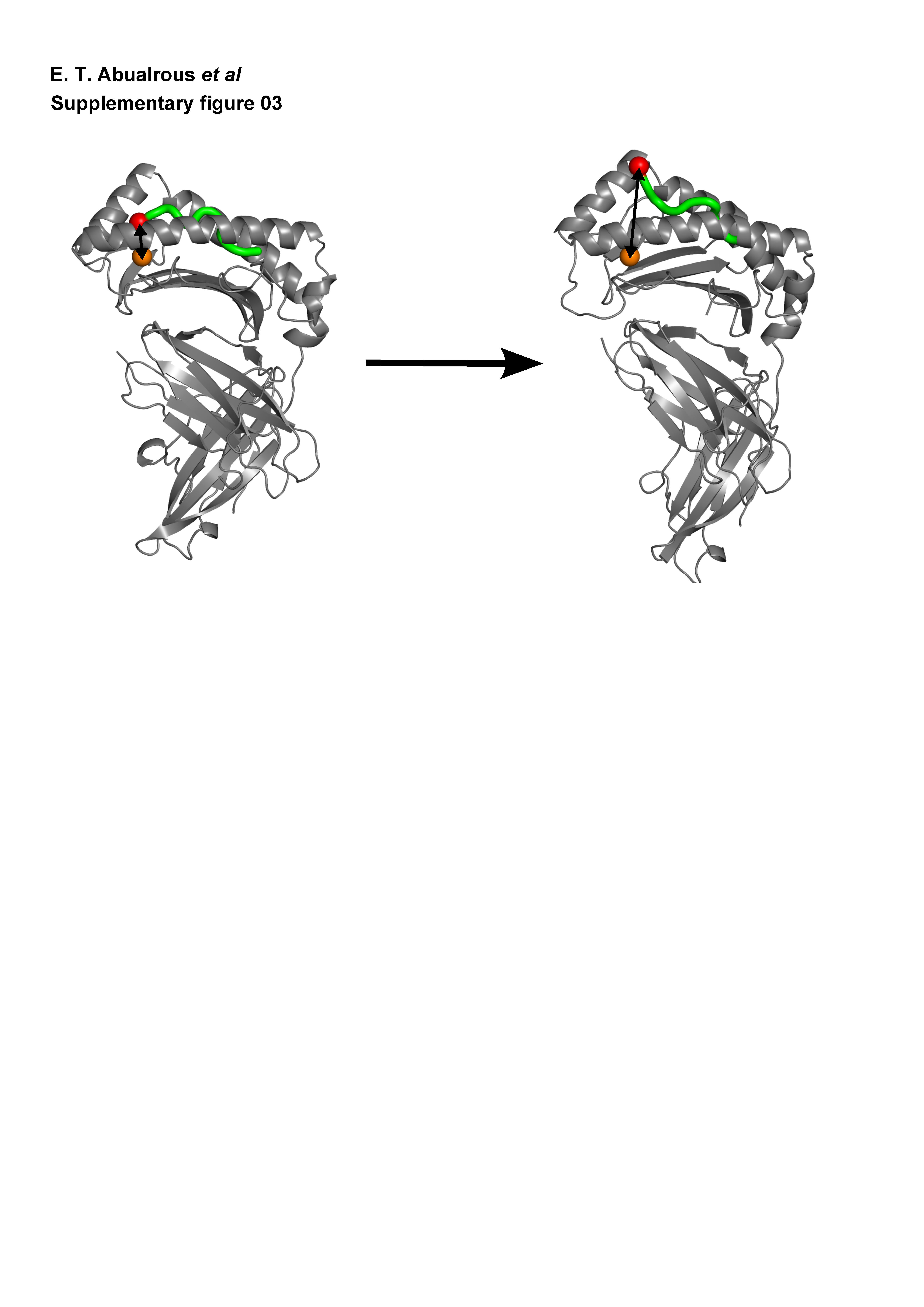
 **S3 Fig. Representative snapshots of the start and end of the reaction coordinates used for the calculations of the free energy change** (potential of mean force, PMF) obtained from umbrella sampling simulations along the distance between the alpha carbon of Pω and the bottom of the F pocket.
