# Supplementary material for: The Carboxy Terminus of the Ligand Peptide Determines the Stability of the MHC Class I Molecule H-2Kb: A Combined Molecular Dynamics and Experimental Study
Source: PLoS One. 2015 Aug 13;10(8):e0135421. doi: 10.1371/journal.pone.0135421 (PMC4535769; doi:10.1371/journal.pone.0135421)
Supplement: S4 Fig — TDTF experiments were performed with the H-2Kb-hβ2m-peptide complex in the presence of different peptide concentration. The Tm values of low-affinity peptide complexes increase with the free peptide concentration, but the relative difference in the Tm values between the C-terminal and N-terminal truncation remains. (DOCX) [file pone.0135421.s004.docx]

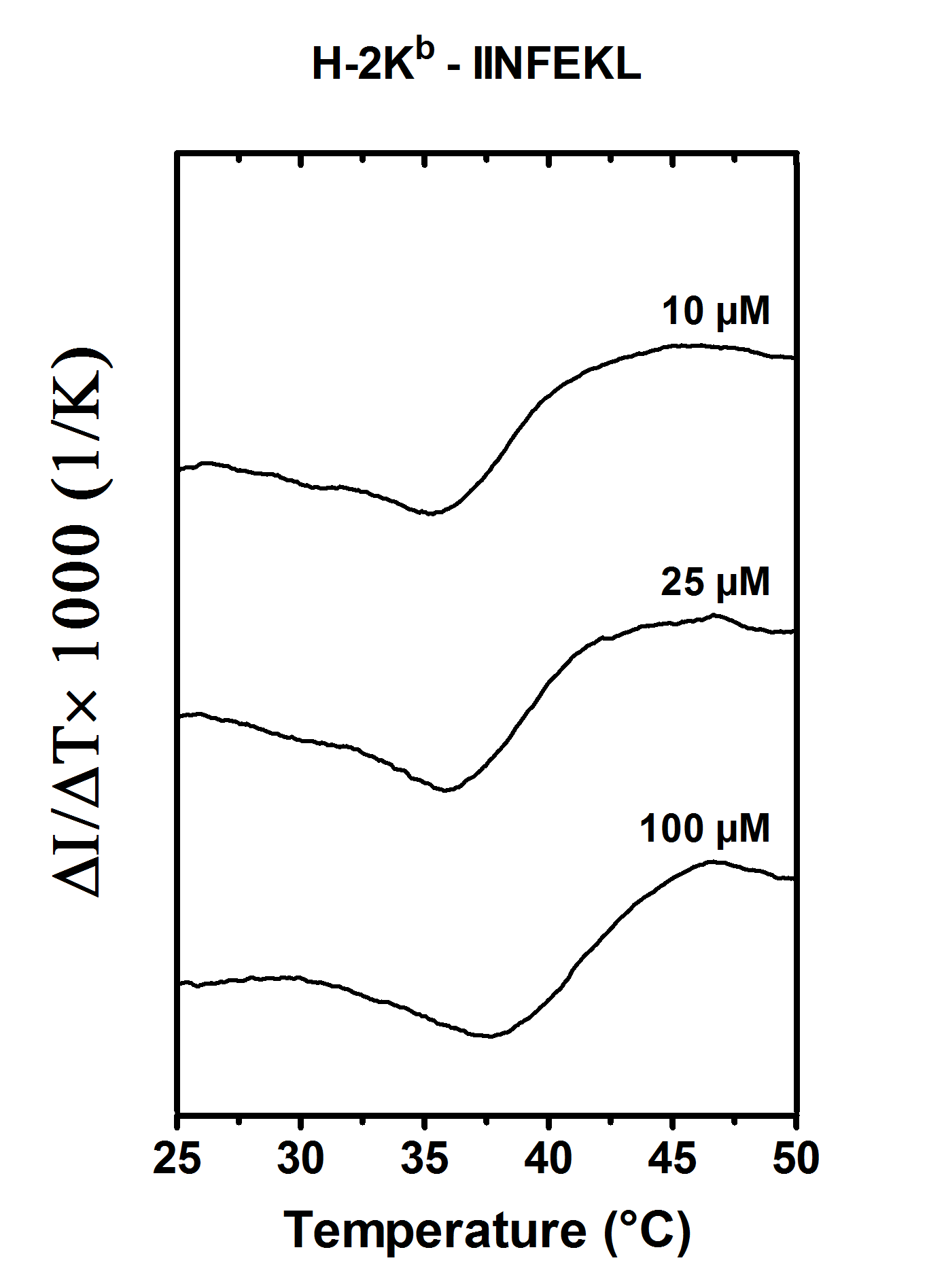

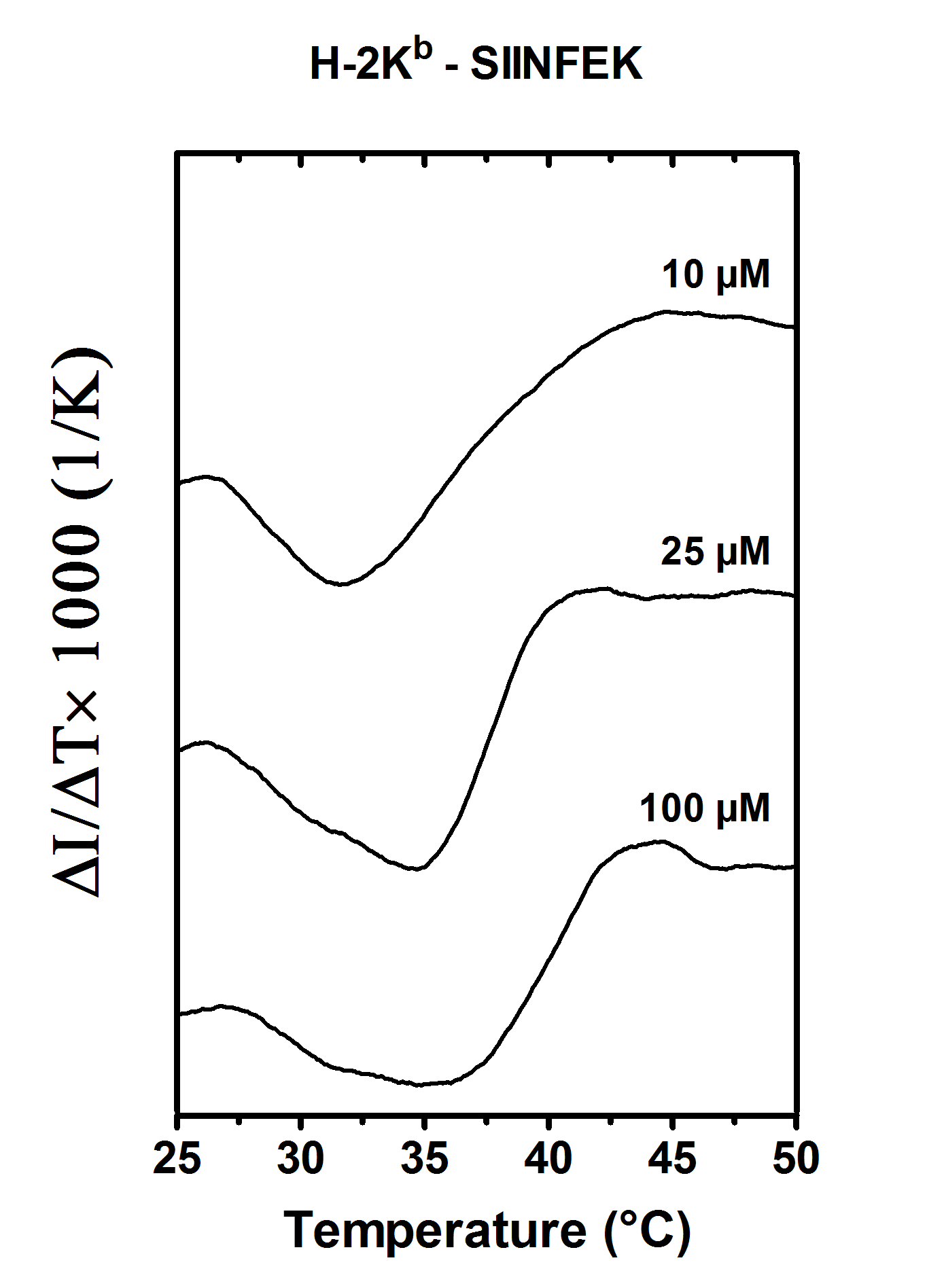


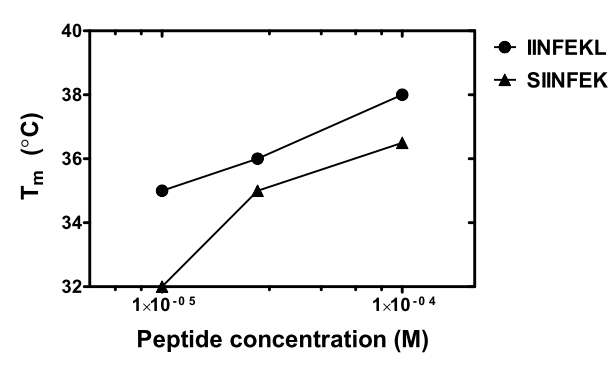


**S4 Fig. Thermal denaturation of low-affinity peptide complexes is dependent of the concentration of free peptide.** TDTF experiments were performed with the H-2K^b^-hβ2m-peptide complex in the presence of different peptide concentration. The T_m_ values of low-affinity peptide complexes increase with the free peptide concentration, but the relative difference in the T_m_ values between the C‑terminal and N‑terminal truncation remains.
