# Supplementary material for: The Carboxy Terminus of the Ligand Peptide Determines the Stability of the MHC Class I Molecule H-2Kb: A Combined Molecular Dynamics and Experimental Study
Source: PLoS One. 2015 Aug 13;10(8):e0135421. doi: 10.1371/journal.pone.0135421 (PMC4535769; doi:10.1371/journal.pone.0135421)
Supplement: S1 Protocol — (DOCX) [file pone.0135421.s005.docx]

**Supporting Information : Detailed Protocols**

**Molecular Dynamics Simulations**

The empty K^b^ structure was prepared by deleting the peptide residues from the crystal structure. The C‑ and N‑terminal truncations and modifications of the peptides were done by deleting or changing the corresponding residues from the peptide structure. For each structure, two independent MD simulations were performed using sander module of Amber 12 simulation package and the parm03 force fields [[1](#_ENREF_1),[2](#_ENREF_2)]. Each complex was initially placed in an octahedral TiP3 [[3](#_ENREF_3)] water box together with ten Na^+^ and Cl^-^ ions and neutralized with eight counter‑ions. Then, it was energy minimized (1500 cycles of steepest descent method), positionally restrained (25 kcal mol^−1^ Å^−2^), and heated from 100 to 300 K. The restraints were resolved in five steps, and then each complex was equilibrated for 1 ns and simulated for 50 ns. Short‑range non bonded interactions were taken into account up to a cut-off value of 9 Å. Long‑range electrostatic interactions were treated with the particle mesh Ewald method (PME), such that the nonbonded interactions (at least the electrostatics) were not truncated at the cut-off [[4](#_ENREF_4)]. Initial velocities were assigned from Maxwellian distribution at the starting temperature. The SHAKE procedure was employed to constrain all hydrogen atoms, and the time step was set to 2 fs [[5](#_ENREF_5)]. RMSD of the backbone atoms of the complex was used to evaluate equilibration. After equilibration, the coordinates of the complexes were saved every 4 ps. Visualization of trajectories and preparation of figures were performed using Visual Molecular Dynamics (VMD) and Pymol [[6](#_ENREF_6),[7](#_ENREF_7)].

Binding energy calculations were performed with the Molecular Mechanics Poisson‑Boltzmann Surface Area tools (MM-PBSA) of the Amber simulation package [[8](#_ENREF_8)]. The single trajectory approach was used to minimize the errors of the calculations [[9](#_ENREF_9),[10](#_ENREF_10)]. The trajectories in which the peptide is bound into the binding groove were clustered according to their RMSD values. Briefly, the trajectories of each complex were clustered into 5 groups based on the measured RMSD of the backbone atoms of the whole molecule. Then, only the trajectories, in which the peptide is bound to the binding groove were included in the analysis to minimize the error values. For each cluster the MM‑PBSA analysis was performed, resulting of 5 values for the binding free energy ∆G of the corresponding peptide. The error values for ∆G of each complex were calculated over the clusters. The energy components were averaged and the standard deviations were calculated over the defined clusters. In MM-PBSA, the binding free energy (∆G_bind_) between the peptide and the empty K^b^ to form K^b^/peptide complex was calculated as

∆G_bind_ = ∆H - T∆S ≈ ∆E_MM_ + ∆G_sol_ −T∆S (1)

∆E_MM_ = ∆E_internal_ + ∆E_electrostatic_ + ∆E_vdw_ (2)

∆G_sol_ = ∆G_PB_ + ∆G_SA_ (3)

where ∆E_MM_, ∆G_sol_, and −T∆S are the changes in the gas phase molecular mechanical energy, solvation free energy, and conformational entropy upon binding. ∆E_MM_ is the sum of ∆E_internal_ (bond, angle, and dihedral energies), ∆E_electrostatic_ (electrostatic energy), and ∆E_vdw_ (van der Waals energy). ∆G_sol_ is the sum of electrostatic solvation energy (polar contribution, ∆G_PB_) and the nonelectrostatic solvation component (nonpolar contribution, ∆G_SA_). The polar contribution is calculated using the Poisson‑Boltzmann model, while the nonpolar energy is estimated by solvent accessible surface area (SASA). The grid spacing is 0.5 Å and the exterior and interior dielectric constants are 80 and 1, respectively. Due to the high computational demand and no significant improvement, the conformational entropy changes (−T∆S) caused by the restriction of the peptide conformation into the bound form were not included in the calculations [[11-15](#_ENREF_11)].

The PMF analysis was performed using umbrella sampling. The K^b^/peptide complexes were simulated with different harmonic distance restraint potentials between the alpha carbon of the C terminal residue of the peptide and the center of mass of residues 95, 97, 116,118, 123, and 124 at the bottom of the F pocket. The biasing potential is given by Equation 4.

W*_i_*(*r*) = *k_i_* (*r* − *r*°)^2^ (4)

where *k_i_* is the force constant, *r* is the reaction coordinate, and *r*° is the reference distance. The equilibrated structure of each complex was used as a starting conformation for the potential of mean force analysis (PMF). A series of 16 (for K^b^/SIINFEKL, K^b^/IINFEKL, and K^b^/SIINFEKA) and 14 (for K^b^/FAPGNYPAL, K^b^/APGNYPAL, and K^b^/FAPGNYPAA) windows was performed. An equilibration of 3 ns was carried out in each window, and the structure obtained at the end of this sampling was used as a starting point for the subsequent window. The reference distance *r*° was increased for each successive window (window step of δ = 0.5 Å) until it reached 18 Å (corresponds to a complete dissociation of the C terminal residue of the peptide).

In each independent window four production MD simulations (2.5 ns) were carried out and force constants *k_i_* are empirically adjusted to ensure significant overlap between adjacent windows in the production run and reach a better convergence. The weighted histogram analysis method (WHAM) was used to yield the unbiased probability distributions of the distance and calculate the PMF or the free energy difference along the reaction coordinates [[16](#_ENREF_16)]. The convergence tolerance for the WHAM calculations of the PMF was equal to 0.001 Å and the error was estimated as the standard deviation of the four production MD sets.

**Thermal denaturation by tryptophan fluorescence (TDTF) measurements:**

The folded heavy chain/β_2_m/peptide complex was prepared by diluting 200 ‏‏µg of the K^b^ heavy chain, 200 ‏‏µg of human β_2_m (both produced in *E. coli)*, and none or 10 µM of peptide into 2 ml of the refolding buffer (100 mM Tris‑Cl pH 8, 0.5 M arginine, 2 mM EDTA, 0.5 mM oxidized glutathione, 5 mM reduced glutathione), and the reaction was incubated at 4 ºC for 48 hours, then ultracentrifuged at 10^5^×g for 20 min to sediment aggregates. Concentration of soluble protein was then determined using the Bradford assay. The yield of folded protein was typically 80%.

The folded complex was measured in PBS (150 mM NaCl, 10 mM phosphate pH 7.4) in a 1 cm × 1 cm cuvette in a Cary Eclipse fluorimeter (Agilent, Waldbronn, Germany) equipped with a Peltier heater and an in-cuvette thermocouple. The sample was heated at 0.30 K/min, and fluorescence values (λex= 290 nm, λem= 345 nm) were recorded every 0.1 K.

The differentials (F(T+∆T) – F(T))/∆T (with F = fluorescence intensity and ∆T = the temperature interval of recording) were smoothed over 40 points. The minimum of the resulting curve allows a simple optical assessment of the T_m_. For display, LOWESS fits [[17](#_ENREF_17)] to the differentials were performed with GraphPad Prism (GraphPad, La Jolla, CA, USA).

The binding free energy components were derived from the TDTF measurements according to the two-state, single-transition model [[18](#_ENREF_18),[19](#_ENREF_19)]. The square of the correlation coefficient (r^2^) was employed to evaluate the correlation between the binding free energies calculated by MM‑PBSA method (∆∆G_MM-PBSA_) and that experimental binding free energies estimated from TDTF measurements ((∆∆G_TDTF_)

**References**

1. D.A. Case TAD, T.E. Cheatham, III, C.L. Simmerling, J. Wang, R.E. Duke, R. Luo, R.C. Walker, W. Zhang, K.M. Merz, B. Roberts, S. Hayik, A. Roitberg, G. Seabra, J. Swails, A.W. Götz, I. Kolossváry, K.F. Wong, F. Paesani, J. Vanicek, R.M. Wolf, J. Liu, X. Wu, S.R. Brozell, T. Steinbrecher, H. Gohlke, Q. Cai, X. Ye, J. Wang, M.-J. Hsieh, G. Cui, D.R. Roe, D.H. Mathews, M.G. Seetin, R. Salomon-Ferrer, C. Sagui, V. Babin, T. Luchko, S. Gusarov, A. Kovalenko, and P.A. Kollman (2012) AMBER 12. University of California, San Francisco.

2. Darden TA, Pedersen LG (1993) Molecular modeling: an experimental tool. Environ Health Perspect 101: 410-412.

3. Jorgensen WL, Chandrasekhar J, Madura JD, Impey RW, Klein ML (1983) Comparison of Simple Potential Functions for Simulating Liquid Water. Journal of Chemical Physics 79: 926-935.

4. Darden T, York D, Pedersen L (1993) Particle Mesh Ewald - an N.Log(N) Method for Ewald Sums in Large Systems. Journal of Chemical Physics 98: 10089-10092.

5. Ryckaert JP, Ciccotti G, Berendsen HJC (1977) Numerical-Integration of Cartesian Equations of Motion of a System with Constraints - Molecular-Dynamics of N-Alkanes. Journal of Computational Physics 23: 327-341.

6. Humphrey W, Dalke A, Schulten K (1996) VMD: visual molecular dynamics. J Mol Graph 14: 33-38, 27-38.

7. DeLano WL "The PyMOL Molecular Graphics System.". DeLano Scientific LLC, San Carlos, CA, USA.

8. Miller BR ea (2012) MMPBSA.py: An efﬁcient program for end-state free energy calculations. J Chem Theory Comput 8: 3314-3321.

9. Hou T, Yu R (2007) Molecular dynamics and free energy studies on the wild-type and double mutant HIV-1 protease complexed with amprenavir and two amprenavir-related inhibitors: mechanism for binding and drug resistance. J Med Chem 50: 1177-1188.

10. Wang JM, Hou TJ, Xu XJ (2006) Recent Advances in Free Energy Calculations with a Combination of Molecular Mechanics and Continuum Models. Current Computer-Aided Drug Design 2: 287-306.

11. Saini SK, Ostermeir K, Ramnarayan VR, Schuster H, Zacharias M, et al. (2013) Dipeptides promote folding and peptide binding of MHC class I molecules. Proc Natl Acad Sci U S A 110: 15383-15388.

12. Muzzioli E, Del Rio A, Rastelli G (2011) Assessing Protein Kinase Selectivity with Molecular Dynamics and MM-PBSA Binding Free Energy Calculations. Chemical Biology & Drug Design 78: 252-259.

13. Xu L, Li YY, Li L, Zhou SY, Hou TJ (2012) Understanding microscopic binding of macrophage migration inhibitory factor with phenolic hydrazones by molecular docking, molecular dynamics simulations and free energy calculations. Molecular Biosystems 8: 2260-2273.

14. Hou TJ, Wang JM, Li YY, Wang W (2011) Assessing the Performance of the Molecular Mechanics/Poisson Boltzmann Surface Area and Molecular Mechanics/Generalized Born Surface Area Methods. II. The Accuracy of Ranking Poses Generated From Docking. Journal of Computational Chemistry 32: 866-877.

15. Genheden S, Kuhn O, Mikulskis P, Hoffmann D, Ryde U (2012) The Normal-Mode Entropy in the MM/GBSA Method: Effect of System Truncation, Buffer Region, and Dielectric Constant. Journal of Chemical Information and Modeling 52: 2079-2088.

16. Kumar S, Bouzida D, Swendsen RH, Kollman PA, Rosenberg JM (1992) The Weighted Histogram Analysis Method for Free-Energy Calculations on Biomolecules .1. The Method. Journal of Computational Chemistry 13: 1011-1021.

17. Cleveland WS, Devlin SJ (1988) Locally Weighted Regression - an Approach to Regression-Analysis by Local Fitting. Journal of the American Statistical Association 83: 596-610.

18. Eftink MR (1994) The Use of Fluorescence Methods to Monitor Unfolding Transitions in Proteins. Biophysical Journal 66: 482-501.

19. Saini SK, Abualrous ET, Tigan AS, Covella K, Wellbrock U, et al. (2013) Not all empty MHC class I molecules are molten globules: Tryptophan fluorescence reveals a two-step mechanism of thermal denaturation. Molecular Immunology 54: 386-396.
