# Supplementary material for: The Carboxy Terminus of the Ligand Peptide Determines the Stability of the MHC Class I Molecule H-2Kb: A Combined Molecular Dynamics and Experimental Study
Source: PLoS One. 2015 Aug 13;10(8):e0135421. doi: 10.1371/journal.pone.0135421 (PMC4535769; doi:10.1371/journal.pone.0135421)
Supplement: S1 Table — The error is calculated as standard deviations over the defined clusters. (DOCX) [file pone.0135421.s006.docx]

**S1 Table.** Experimental and calculated binding free energy (kcal/mol) using MM-PBSA and TDTF methods for H‑2K^b^/peptide complexes. The error is calculated as standard deviations over the defined clusters.

|  | **∆E_vdw_** | **∆E_elec_** | **∆G_PB_** | **∆G_SA_** | **∆G_MM-PBSA_** | **∆∆G_MM-PBSA_** | **∆G_TDTF_** | **∆∆G_TDTF_** |
| --- | --- | --- | --- | --- | --- | --- | --- | --- |
| **SIINFEKL** | -77.5 ±4.1 | -222.0 ±16.3 | 249.6 ±12.7 | -8.85 ±0.5 | -58.7 ±7.2 | 0 | -104.5 ±10.4 | 0 |
| **-IINFEKL** | -66.6 ±2.4 | -199.0 ±19.2 | 233.4 ±15.4 | -7.95 ±0.4 | -40.5 ±2.9 | 18.2 | -65.3 ±6.5 | 39.2 |
| **SIINFEK-** | -61.1 ±2.2 | -207.2 ±13.3 | 244.0 ±10.0 | -6.65 ±0.2 | -30.7 ±3.3 | 28.0 | -57.9 ±5.7 | 46.6 |
| **SIINFEKA** | -70.1 ±6.0 | -218.1 ±9.6 | 245.6 ±8.3 | -7.45 ±0.3 | -50.0 ±5.4 | 8.8 | -92.8 ±9.3 | 11.8 |
| **SIINFEKL-Cdel** | -73.4 ±6.5 | -182.6 ±14.2 | 224.4 ±6.3 | -7.75 ±0.6 | -39.3 ±2.7 | 19.4 | -83.8 ±8.3 | 20.7 |
| **SIINFEKG** | -69.7 ±4.2 | -220.0 ±13.7 | 245.7 ±13.4 | -7.05 ±0.6 | -51.0 ±4.9 | 7.7 | -92.6 ±9.2 | -11.9 |
| **FAPGNYPAL** | -75.2 ±4.5 | -210.7 ±16.3 | 242.4 ±15.2 | -8.9 ±0.6 | -51.6 ±1.9 | 0 | -121.1 ±12.1 | 0 |
| **-APGNYPAL** | -67.0 ±6.0 | -205.5 ±17.8 | 247.2 ±11.1 | -7.7 ±0.4 | -32.9 ±2.6 | 18.8 | -81.8 ±8.2 | 39.3 |
| **FAPGNYPA-** | -56.4 ±1.1 | -204.2 ±13.3 | 244.1 ±10.0 | -7.3 ±0.3 | -23.6 ±1.7 | 27.0 | -65.8 ±6.6 | 55.4 |
| **FAPGNYPAA** | -71.0 ±1.7 | -208.6 ±12.5 | 246.3 ±8.3 | -7.5 ±0.1 | -40.6 ±1.6 | 11.0 | -97.0 ±9.7 | 24.1 |
