# Supplementary material for: The Carboxy Terminus of the Ligand Peptide Determines the Stability of the MHC Class I Molecule H-2Kb: A Combined Molecular Dynamics and Experimental Study
Source: PLoS One. 2015 Aug 13;10(8):e0135421. doi: 10.1371/journal.pone.0135421 (PMC4535769; doi:10.1371/journal.pone.0135421)
Supplement: S2 Table — The error is calculated as standard deviation. (DOCX) [file pone.0135421.s007.docx]

**S2 Table.** Thermal denaturation measured by tryptophan fluorescence (TDTF) shows the T_m_ of K^b^/β_2_m empty or in complex with peptide, as indicated. The error is calculated as standard deviation.

|  | **T_m_ (°C)** |
| --- | --- |
| **empty** | 33.0 ±1.16 |
| **SIINFEKL** | 55.7 ±0.58 |
| **-IINFEKL** | 37.7 ±0.58 |
| **SIINFEK-** | 33.4 ±1.15 |
| **SIINFEKA** | 44.4 ±0.58 |
| **FAPGNYPAL** | 51.3 ±0.58 |
| **-APGNYPAL** | 40.7 ±0.58 |
| **FAPGNYPA-** | 33 ±1.41 |
| **FAPGNYPAA** | 41.3 ±1.15 |
